# Metabolomics approach to identify key volatile aromas in Thai colored rice cultivars

**DOI:** 10.3389/fpls.2023.973217

**Published:** 2023-02-28

**Authors:** Rossarin Tansawat, Supawat Jindawatt, Paweena Ekkaphan, Siriphat Ruengphayak, Apichart Vanavichit, Nitima Suttipanta, Sornkanok Vimolmangkang, Wanchai De-Eknamkul

**Affiliations:** ^1^Department of Food and Pharmaceutical Chemistry, Faculty of Pharmaceutical Sciences, Chulalongkorn University, Bangkok, Thailand; ^2^Scientific and Technological Research Equipment Center, Chulalongkorn University, Bangkok, Thailand; ^3^Rice Science Center & Rice Gene Discovery Unit, Kasetsart University, Nakhon Pathom, Thailand; ^4^Department of Agronomy, Faculty of Agriculture at Kamphaeng Saen, Kasetsart University, Nakhon Pathom, Thailand; ^5^Faculty of Pharmaceutical Sciences, Ubon Ratchathani University, Ubon Ratchathani, Thailand; ^6^Department of Pharmacognosy and Pharmaceutical Botany, Faculty of Pharmaceutical Sciences, Chulalongkorn University, Bangkok, Thailand

**Keywords:** colored rice, black rice, volatile, aroma, metabolomics, headspace, GC-MS

## Abstract

In addition to white jasmine rice, Thailand has many native-colored rice varieties with numerous health benefits and the potential to become a global economic crop. However, the chemical characteristics of aromatic substances in native-colored rice are still mostly unknown. This study aimed to identify the key volatile aroma compounds and the biosynthetic pathways possibly involved in their formation in Thai native-colored rice varieties, and thus leading to the search for potential genetic markers for breeding colored rice with better aromatic properties. Twenty-three rice varieties in four categories: aromatic white, aromatic black, non-aromatic black, and non-aromatic red, were investigated (n=10 per variety). Seed husks were removed before the analysis of rice volatile aromas by static headspace gas chromatography–mass spectrometry. Untargeted metabolomics approach was used to discover the key volatile compounds in colored rice. Forty-eight compounds were detected. Thirty-eight of the 48 compounds significantly differed among groups at p<0.05, 28 of which at p<0.0001, with the non-aromatic black and red rice containing much lower content of most volatile constituents than the aromatic black and white rice. Focusing on the aromatic black rice, the samples appeared to contain high level of both compound groups of aldehydes (3-methylbutanal, 2-methylbutanal, 2-methylpropanal, pentanal, hexanal) and alcohols (butane-2,3-diol, pentan-1-ol, hexan-1-ol). Biosynthetically, these distinctive black-rice volatile compounds were proposed to be formed from the metabolic degradation of branched-chain amino acids (L-leucine, L-isoleucine and L-valine) and polyunsaturated fatty acids (linoleic acid and α-linolenic acid), involving the branched-chain aminotransferases and keto-acid decarboxylases and the 9-lipoxygonases and 13-lipoxygeases, respectively. The proposed degradative pathways of amino acids and fatty acids were well agreed with the profiles key volatile compounds detected in the Thai native-colored rice varieties.

## 1 Introduction

In addition to the well-known white jasmine rice, Thailand has numerous rice varieties with potential to become a worldwide economic crop. Consumers are currently interested in colored rice because of its health benefits (Vanavichit, 2022), particularly its antioxidant effects, stronger than in white rice (Walter et al., 2013; Sansenya and Nanok, 2020). Thai native-colored rice such as riceberry, black glutinous rice, red rice, etc., have dark tones ranging from red, brown, and black due to the accumulation of proanthocyanin, anthocyanin, flavonoid, and phenolic acid compounds. Antioxidant activity, anti-hyperlipidemia, oxidative stress reduction, and anti-carcinogenic activity have all been related to the anthocyanins present in colored rice (Sivamaruthi et al., 2018).

Aroma and flavor are especially important factors in determining the quality and character of rice, as well as the consumer preference. It has been reported that there are pronounced differences in aroma between black and white rice (Yang et al., 2008). Chemically, more than 200 volatile compounds have been found in cooked rice of various varieties (Jezussek et al., 2002), which can be classified into seven groups: hydrocarbons, aldehydes, alcohols, ketones, acids, esters, and heterocyclic compounds (Hashemi et al., 2013). Among these, hydrocarbons and aldehydes account for the greatest proportion by weight of volatile constituents in rice (Lin et al., 2010). The hydrocarbons found in rice include 2,6,10-trimethyldodecane, pentadecane, 2,6,10-trimethylpentadecane, hexadecane, and heptadecane, while the aldehydes include pentanal, hexanal, heptanal, 2-heptene aldehyde, octanal, nonanal, decyl aldehyde, 2-methypropanal, 2-methylbutanal, 3-methylbutanal and benzene formaldehyde (Yang et al., 2008; Lin et al., 2010; Zheng et al., 2022). Among alcohols, 1-octen-3-ol, hexanol, and 1-octanol have been considered to be more abundant compounds than the aldehydes (Yang et al., 2008). The most representative fragrance for identifying the overall aroma of rice is 2-acetyl-1-pyrroline (2-AP) which belongs to the pyrroline class of compounds (Wei et al., 2017). However, 2-AP is not the only component distinguishing between aromatic rice and non-aromatic rice. The mentioned hydrocarbons, aldehydes, alcohols and heterocyclics also played important role in rice aroma quality (Hu et al., 2020). In terms of physiological functions, the information on the roles of the volatile compounds in rice is still limited. However, it has been shown that rice volatile compounds can be induced by brown planthoppers (Jannoey et al., 2016), and thus probably functioning as plant defense compounds against insects. Genetically, the main candidate gene that has been proposed to contribute to the aroma in rice is fgr/badh2/Os2AP (Routray and Rayaguru, 2018). This gene is involved in the biosynthetic pathway of 2-AP and homologous to betaine aldehyde dehydrogenase (BADH) located on chromosome 8. However, there are other unidentified genes, especially those involved in the biosynthesis of the volatile compounds, contributed to the aroma in rice (Hu et al., 2020).

Moreover, according to Kushwaha (2016), colored rice is high in fiber and protein but low in carbohydrate, making it ideal as plant-based food. Black rice in particular, has a higher protein but lower carbohydrate content than other rice varieties (Kushwaha, 2016). For a healthy diet and sustainable food production, increasing the consumption of plant-based diets and less animal-based foods is key (Päivärinta et al., 2020; Langyan et al., 2021). The global plant-based food market is predicted to grow from 29.4 billion USD in 2020 to 161.9 billion USD in 2030 (Statista, 2022).

Currently, Thailand has developed novel colored rice types, both nutritious and appealing to customers (Vanavichit, 2022). Although their aromatic properties have been established in several colored rice cultivars, information on the chemical characteristics of volatile substances in these colored rice varieties is still limited, particularly the compounds responsible for colored rice’s distinct scent and flavor compared to white rice’s. Hence, this study aimed to analyze the types of volatile compounds in Thai native-colored rice varieties and determine key volatile compounds which could indicate biosynthesis pathways and genetic markers for improvement of Thai colored rice.

The technology for analyzing the candidate marker compounds has tremendously come out over the years. Untargeted volatile metabolomics, the hypothesis-generating tool (Schrimpe-Rutledge et al., 2016), is an emerging technique that combines high-resolution technology, like mass spectrometry or nuclear magnetic resonance, with advanced statistical analysis to extract the important compounds among a large number of metabolites in a biological sample. Metabolomics analysis of volatile organic compounds is applied in various research fields, notably medicine (Sukaram et al., 2022), food (Diez-Simon et al., 2019), and plant sciences (Mhlongo et al., 2022). In this study, static headspace gas chromatography–mass spectrometry (SHS-GC-MS) was employed for the analysis of the volatile compounds in rice samples. Based on the key volatile aromas identified by statistical analysis, the biosynthetic pathways responsible for certain chemicals were postulated. Such biosynthetic processes consist of biochemical steps due to the action of various related enzymes. This biosynthetic data would help reveal target gene groups potentially used as genetic markers for improvement of volatile colored rice breeding.

## 2 Materials and methods

### 2.1 Rice plants

Colored rice varieties that have the same allele of Os2AP as reference aromatic white rice, Khao Dawk Mali 105 (KDML105), were selected for this study. Twenty-three rice varieties in four categories: aromatic white, aromatic black, non-aromatic black, and non-aromatic red (**Table 1**), were planted at the Rice Science Center, Kasetsart University, Kamphaeng Sean Campus, Nakhon Pathom Province, Thailand, during 2018’s wet season (August 2018- January 2019). Twenty-one-day-old seedlings were transplanted to the paddy field at 10 rows x 10 plants per row per variety, with 25 X 25 cm plant spacing and 50 cm variety spacing. Paddy seeds from each variety were harvested from 10 randomly selected plants.

**Table 1:** List of rice samples.

No.	Rice varieties	Code	Pericarp color	BADH2 allele
1	Basmati 370	BMT	white	aromatic
2	Khao Dawk Mali 105	KDML105	white	aromatic
3	Klamhom	KH	black	aromatic
4	LeumPua glutinous rice	LP	black	aromatic
5	UP_460_Chanohnai	UP_460	black	aromatic
6	UP_463_Pi-isu	UP_463	black	aromatic
7	UP_468_Pi-isu Maeradnoi	UP_468	black	aromatic
8	UP_469_Pi-isu Maekwangnuea	UP_469	black	aromatic
9	UP_470_Pi-isu Maekwangnuea	UP_470	black	aromatic
10	Niew Dam khaika glutinous rice	DKG	black	aromatic
11	Khao Hom Mae Phaya Tongdam	MTK	black	aromatic
12	Mu1309	Mu1309	black	aromatic
13	Mu2313	Mu2313	black	aromatic
14	Mu2550	Mu2550	black	aromatic
15	Riceberry 2 (#909)	RB2	black	aromatic
16	Niew Dam Chomaipai 49 glutinous rice	BSHMP	black	non-aromatic
17	Riceberry	RB	black	non-aromatic
18	Niew Dammo 37 glutinous rice	DM37	black	non-aromatic
19	Niew Dammuebueng glutinous rice	DMB	black	non-aromatic
20	Jao Hom Nin	JHN	black	non-aromatic
21	Khao Mednaifuy	MNF	black	non-aromatic
22	RD 69 (Tubtim Chumphae)	RUBY	red	non-aromatic
23	UP_417_Buetolasosobkhong	UP_417	red	non-aromatic

### 2.2 Seed preparation

The husk of paddy rice seed was removed by hand. One hundred and ten seeds per plants and 10 plants per variety from each field location were collected and stored at -80°C before analysis.

### 2.3 Metabolomic analysis

#### 2.3.1 Sample preparation

Rice sample preparation was done using the optimum condition previously established by Jindawatt et al. (2021), which specifically developed for the volatile analysis of colored rice. Briefly, 1 g of rice was placed into 10-mL headspace vials. Then, 10 µL 99% 2,4,6-trimethylpyridine (CAS No. 108-75-8, Alfa Aesar, Heysham, England) was added into the vials as internal standard. The vials were sealed and preheated in a hot air oven (WTC Binder Bd-53, Tuttlingen, Germany) at 80°C for 5 h before being transferred to the SHS-GC-MS in order to extract as many volatile compounds as possible.

#### 2.3.2 Volatile compound analysis

After equilibration, SHS-GC-MS analysis was carried out with a 7697A SHS autosampler coupled to 7890B GC system and 7000C QQQ MS (Agilent Technologies, Palo Alto, CA, USA) equipped with an HP-5ms capillary column (5% phenyl/95% dimethylpolysiloxane, 30 m × 0.25 mm i.d., 0.25 μm film thickness, Agilent, CA, USA). A single quadrupole in scan mode was used for GC-MS analysis, which suitable for qualitative analysis or identification (an untargeted metabolomics). Samples were placed into a headspace autosampler oven and incubated again at 120°C for 60 min. Next, 1-mL headspace volatile was collected at 140°C and directly introduced into a GC-MS system. The temperature of the GC inlet was 220°C. Ultra-high purity helium (99.99%) was used as carrier gas at average velocity of 35 cm/s and a 20:1 split ratio. The initial oven temperature was set at 40°C for 2 min, ramped to 250°C at 5°C/min, and finally held for 4 min. The MS was operated in electron impact (EI) mode at 70 eV. The temperature of the MS interface, EI source, and quadrupole were set at 250, 230, and 150°C, respectively. Chromatogram and mass spectra were acquired using a scan mode ranging from 33-400 m/z.

### 2.4 Data processing and statistical analysis

Rice volatile compounds were identified by comparing both the mass spectra and retention index (RI) against the National Institute of Standards and Technology library (NIST) 2014 library. The RI of the n-alkane series (C7-C30; Supelco, Sigma-Aldrich, PA, USA) was used to compute the RI values. A matching score ≥70 and a RI value difference ≤20 units between the calculated RI and the database values for the same stationary phase were required for compound identification.

A pooled quality control (QC) sample was included every 10 samples. Peak picking, spectral deconvolution, and data alignment were performed using MS-DIAL 4.70 software (Tsugawa et al., 2015). Multivariate analysis, metabolite set enrichment analysis (MSEA), and analysis of variance (ANOVA) were performed with MetaboAnalyst 5.0 (Xia et al., 2015).

## 3 Results

**Figure 1:**
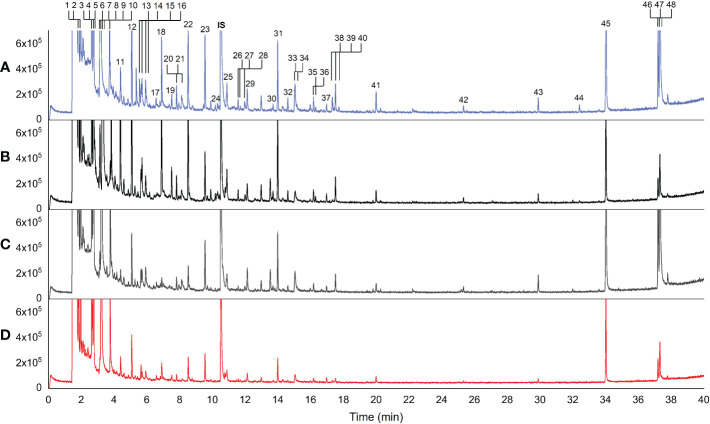
Representative chromatograms of rice volatile compounds detected by static headspace gas chromatography–mass spectrometry method. **(A)** aromatic white rice (Basmati 370), **(B)** aromatic black rice (Klamhom), **(C)** non-aromatic black rice (Riceberry), **(D)** non-aromatic red rice (RD 69 Tubtim Chumphae); IS = internal standard. Each number represented the identified volatile compounds arranged by retention time.

Forty-eight volatile compounds were identified in 23 rice varieties (**Table 2**; **Figure 1**). **Figure 1** displays representative chromatograms from the four rice groups along with numbers (1-48) sorted by retention time (RT). The numbers indicated the identified volatile compounds shown in **Table 2**. According to the Metabolomics Standards Initiative guidelines (Sumner et al., 2007), the volatile compounds identified in this study were MSI level 2 (putative annotated compounds). Statistical analyses by ANOVA followed by Tukey’s HSD *post-hoc* test showed significant differences at p<0.05 in 38 of the 48 compounds among the four rice groups, 28 of which at p<0.0001 (**Table 2**). A complete dataset is presented in **Supplementary Table 1**, which includes retention time (RT), RI, metabolite ID, formula, MS fragment pattern (m/z), CAS no., InChIKey, matching score, signal-to-noise ratio (S/N), EI spectrum, and peak area.

**Table 2:** Volatile compounds detected in aromatic white, aromatic black, non-aromatic black, and non-aromatic red rice samples.

No.	RT (min)	Metabolite name	Formula	Identification	MS fragment pattern (m/z)	p-value*
1	1.829	methyl acetate	C_3_H_6_O_2_	MS, RI	**43**, 74, 42, 59, 44, 45, 41	NS
2	1.961	2-methylpropanal	C_4_H_8_O	MS, RI	43, 41, 72, **39**, 42, 38	<0.0001
3	2.641	3-methylbutanal	C_5_H_10_O	MS, RI	**44**, 43, 41, 58, 39, 57, 71, 42	<0.0001
4	2.746	2-methylbutanal	C_5_H_10_O	MS, RI	41, **57**, 58, 39, 43, 86, 55	NS
5	2.818	4-(dimethylamino)-3-hydroxybutanoic acid	C_6_H_13_NO_3_	MS, RI	**58**, 42, 44, 88, 59, 147, 33, 43	NS
6	3.107	pentane-2,3-dione	C_5_H_8_O_2_	MS, RI	**43**, 57, 42, 100	NS
7	3.151	pentanal	C_5_H_10_O	MS, RI	**44**, 58, 41, 57, 43, 39, 42, 45	NS
8	3.232	acetic acid	C_2_H_4_O_2_	MS, RI	43, 45, **60**, 42, 41	NS
9	3.310	formyl acetate	C_3_H_4_O_3_	MS, RI	43, 45, 44, 42, **60**, 87	<0.001
10	3.731	1-hydroxypropan-2-one	C_3_H_6_O_2_	MS, RI	**43**, 74, 42, 45, 44	NS
11	4.390	pentan-1-ol	C_5_H_12_O	MS, RI	42, **55**, 41, 70, 43, 57, 39	<0.001
12	5.075	hexanal	C_6_H_12_O	MS, RI	44, 56, 41, 43, 57, 39, 45, **72**, 82	<0.0001
13	5.568	butane-2,3-diol	C_4_H_10_O_2_	MS, RI	**45**, 43, 57, 47, 44, 46	NS
14	5.698	4-methylpyrimidine	C_5_H_6_N_2_	MS, RI	**94**, 40, 53, 67, 39, 52, 79, 38	<0.0001
15	5.927	furan-2-carbaldehyde	C_5_H_4_O_2_	MS, RI	**96**, 95, 39, 67, 38, 98, 43, 54	<0.0001
16	5.984	1-(5-methyl-1H-pyrazol-3-yl)propan-2-amine	C_7_H_13_N_3_	MS, RI	**43**, 95, 96, 39, 42, 87, 41, 29	<0.0001
17	6.628	3,3-dimethyl-4-(methylamino)butan-2-one	C_7_H_15_NO	MS, RI	**60**, 41, 43, 42, 40, 39, 29, 45	<0.01
18	6.901	hexan-1-ol	C_6_H_14_O	MS, RI	**56**, 43, 41, 55, 39, 69, 84	<0.0001
19	7.505	heptan-2-one	C_7_H_14_O	MS, RI	**43**, 58, 71, 41, 39, 59, 42, 99, 114	<0.0001
20	7.799	heptanal	C_7_H_14_O	MS, RI	**70**, 41, 44, 43, 55, 57, 42, 39, 81, 96	<0.0001
21	8.116	2,6-dimethylpyrazine	C_6_H_8_N_2_	MS, RI	**108**, 42, 40, 39, 38, 41, 67, 109, 37	<0.0001
22	8.514	methyl hexanoate	C_7_H_14_O_2_	MS, RI	**74**, 87, 43, 59, 99, 55, 41, 101, 42, 71	<0.0001
23	9.539	benzaldehyde	C_7_H_6_O	MS, RI	77, **106**, 105, 51, 50, 78, 52, 74, 107, 39	<0.0001
24	10.307	2-propylpropanedioic acid	C_6_H_10_O_4_	MS, RI	**60**, 44, 73, 41, 43, 45, 55, 42	<0.05
25	10.881	octanal	C_8_H_16_O	MS, RI	43, 44, 41, 56, **84**, 57, 55, 42, 69, 100	<0.0001
26	11.563	methyl 5-methylhexanoate	C_8_H_16_O_2_	MS, RI	74, 87, **43**, 55, 113, 40, 41, 29	<0.0001
27	11.582	methyl heptanoate	C_8_H_16_O_2_	MS, RI	**74**, 87, 43, 113, 55, 101, 59, 41, 39, 75	<0.0001
28	11.976	3-hydroxy-4,4-dimethyloxolan-2-one	C_6_H_10_O_3_	MS, RI	**71**, 43, 41, 57, 55, 39, 72, 56	<0.01
29	12.117	2-phenylacetaldehyde	C_8_H_8_O	MS, RI	**91**, 92, 120, 65, 39, 63, 51, 89, 121, 50	<0.05
30	13.515	2-methoxyphenol	C_7_H_8_O_2_	MS, RI	109, **124**, 81, 53, 52, 51, 39, 50, 63, 110	<0.0001
31	13.977	nonanal	C_9_H_18_O	MS, RI	**57**, 41, 43, 56, 44, 55, 70, 98, 69	<0.01
32	14.598	methyl octanoate	C_9_H_18_O_2_	MS, RI	**74**, 87, 43, 41, 55, 57, 127, 59, 115	<0.0001
33	15.038	methyl pyridine-3-carboxylate	C_7_H_7_NO_2_	MS, RI	**106**, 78, 137, 136, 51, 50, 138, 107	<0.01
34	15.150	1-methylpyridin-1-ium-3-carboxylate	C_7_H_7_NO_2_	MS, RI	**106**, 78, 137, 95, 68, 40, 151, 135	<0.0001
35	16.165	methyl 2-phenylacetate	C_9_H_10_O_2_	MS, RI	**91**, 150, 65, 92, 89, 59, 63, 39, 90, 151	<0.0001
36	16.286	naphthalene	C_10_H_8_	MS, RI	**128**, 129, 127, 51, 64, 102, 126, 63, 77, 75	<0.0001
37	16.962	decanal	C_10_H_20_O	MS, RI	43, 41, **57**, 55, 44, 70, 56, 68, 71, 112	<0.01
38	17.324	2,3-dihydro-1-benzofuran	C_8_H_8_O	MS, RI	**120**, 91, 119, 92, 39, 89, 63, 65, 121, 51	<0.0001
39	17.496	methyl nonanoate	C_10_H_20_O_2_	MS, RI	**74**, 87, 55, 43, 41, 59, 141, 129, 143, 57	<0.0001
40	17.732	3-ethyl-4-methylpyrrole-2,5-dione	C_7_H_9_NO_2_	MS, RI	53, **139**, 67, 68, 124, 96, 110, 94, 95, 111	<0.0001
41	19.973	1-(2-hydroxy-5-methylphenyl)ethanone	C_9_H_10_O_2_	MS, RI	135,**150**, 107, 77, 43, 136, 51, 151, 79, 39	<0.0001
42	25.311	methyl 10-methylundecanoate	C_13_H_26_O_2_	MS, RI	**74**, 87, 57, 41, 43, 55, 69, 143, 59, 75, 214	<0.0001
43	29.865	methyl 12-methyltridecanoate	C_15_H_30_O_2_	MS, RI	**74**, 87, 43, 55, 41, 199, 57, 143, 59, 75	NS
44	32.394	6,10,14-trimethylpentadecan-2-one	C_18_H_36_O	MS, RI	**43**, 58, 71, 57, 59, 41, 55, 69, 85, 95, 250	<0.0001
45	34.001	methyl hexadecanoate	C_17_H_34_O_2_	MS, RI	**74**, 87, 43, 55, 41, 143, 75, 57, 69, 227, 270	<0.001
46	37.165	methyl (9Z,11E)-octadeca-9,11-dienoate	C_19_H_34_O_2_	MS, RI	67, 81, 95, 79, **55**, 82, 96, 68, 109, 69, 294	NS
47	37.185	methyl (10E,12Z)-octadeca-10,12-dienoate	C_19_H_34_O_2_	MS, RI	67, **81**, 95, 55, 82, 79, 96, 68, 294, 54	<0.01
48	37.296	methyl-octadec-9-enoate	C_19_H_36_O_2_	MS, RI	**55**, 69, 74, 83, 97, 41, 96, 87, 43, 84, 222, 264, 296	<0.0001

*P-value is determined by ANOVA among the four groups of rice (aroma white, aroma black, non-aroma black, and non-aroma red rice) of each compound; NS, not significant (p>0.05).

MS, mass spectra; RI, retention index. Bold values mean the most ion abundant of each compound.

**Figure 2:**
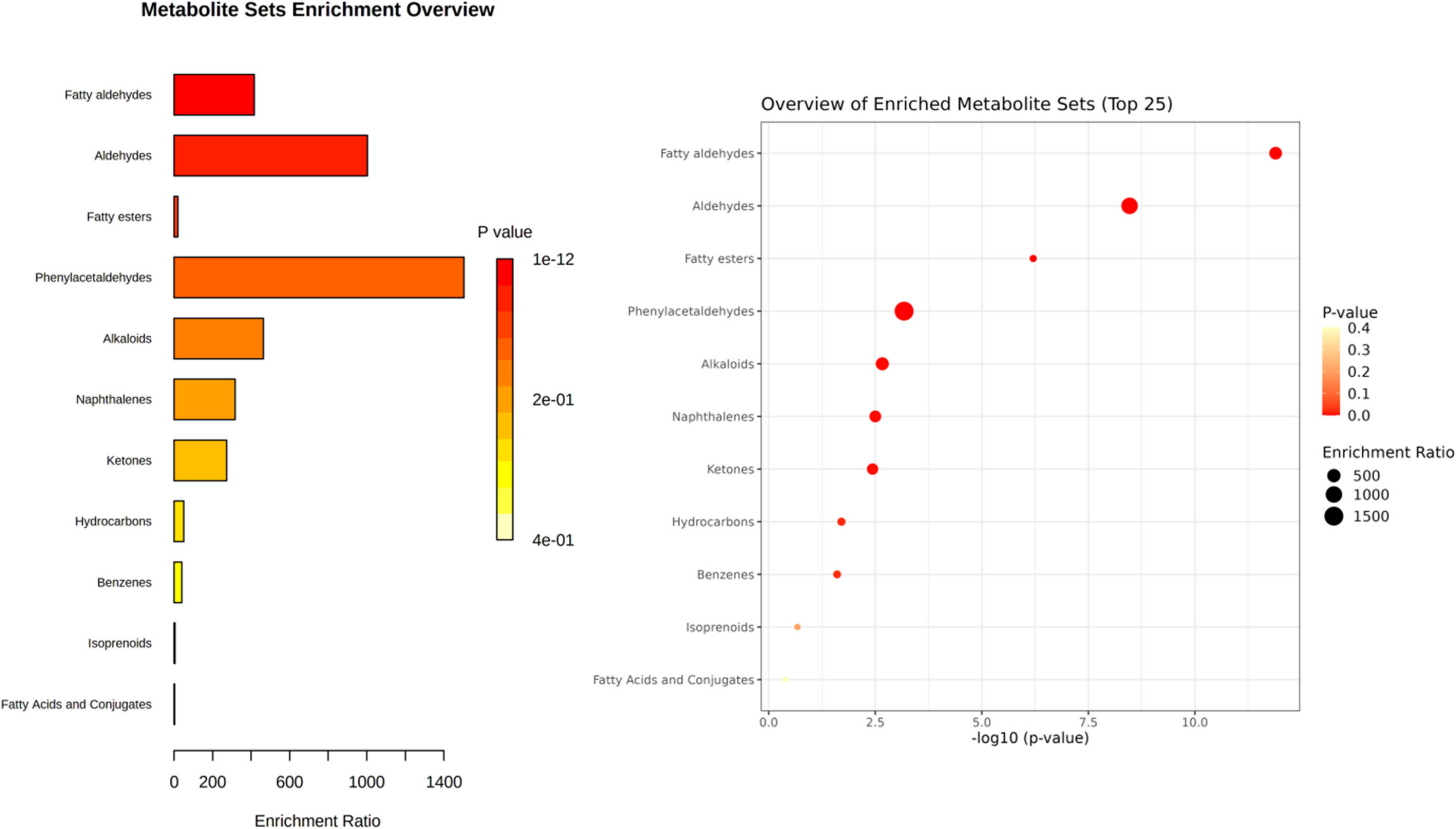
Overview of volatile compound groups identified from 23 Thai rice varieties (aromatic white rice, aromatic black rice, non-aromatic black rice, and non-aromatic red rice). Metabolite sets (volatile compounds) are classified by sets of main-class chemicals. The bar chart’s colors are determined by the p-value. For dot plot, each color and size are determined by p-value and the enrichment ratio, respectively.

MSEA was carried out to observe the patterns of the main chemical class sets by MetaboAnalyst software. In the MetaboAnalyst 5.0 database, 33 out of the 48 chemicals identified had a PubChem CID (compound ID number) match, as shown in the overview of aroma compound sets in **Figure 2**. Colors of the bar chart are based on p-value. For dot plot, the color and size of each circle are based on p-value and the enrichment ratio, respectively. **Figure 2** shows that fatty aldehydes, aldehydes, and fatty esters were the most common volatile chemical classes found in the 23 Thai rice varieties.

**Figure 3:**
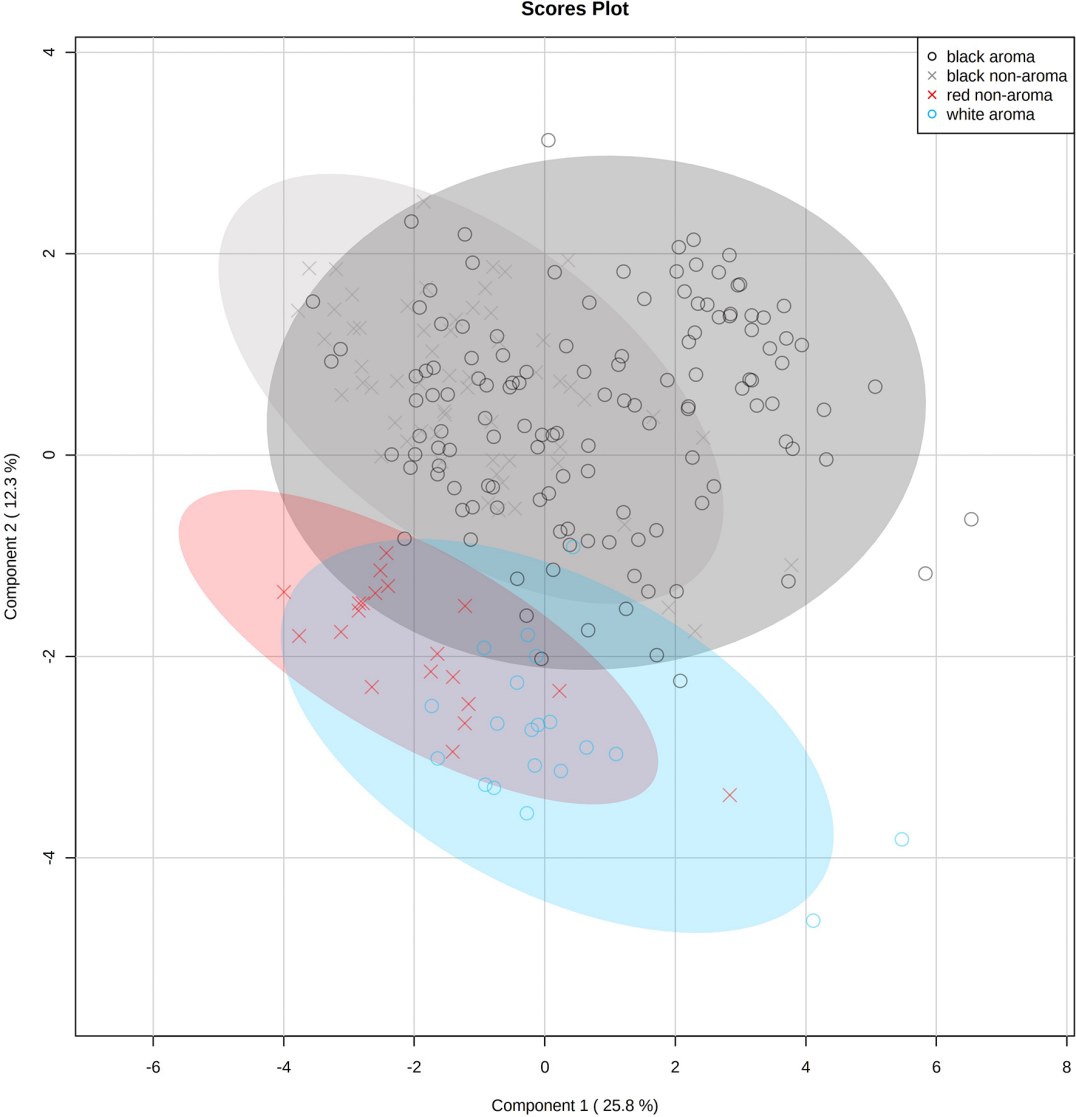
Partial least squares-discriminant analysis (PLS-DA) scores plot of volatile profiles of 23 Thai rice varieties (aromatic white, aromatic black, non-aromatic black, and non-aromatic red rice samples) identified by static headspace gas chromatography–mass spectrometry technique. One replication is represented by each symbol (n=10 per rice variety), while 95% confidence intervals are represented by shaded circles.

Prior to multivariate statistical analysis, data were normalized using log transform and pareto scaled for volatile chemical profiling. A partial least squares-discriminant analysis (PLS-DA) was carried out to determine the differences among the four groups of rice. R^2^ = 0.76 and Q^2^ = 0.68 according to the model, indicating the goodness of fit and predictability, respectively. A permutation test was used to check whether the PLS-DA models were overfitted (see **Supplementary Figure S1** for the output of permutation test). PLS-DA scores plot shows different volatile profiles among the four rice types (**Figure 3**). One sample replication is represented by each symbol whereas shaded circles indicate 95% confidence intervals. White and black rice patterns, and black and red rice samples, are the opposite. The red rice volatile profile tends to resemble that of white rice. However, the aromatic and non-aromatic black rice profiles are remarkably similar.

**Figure 4:**
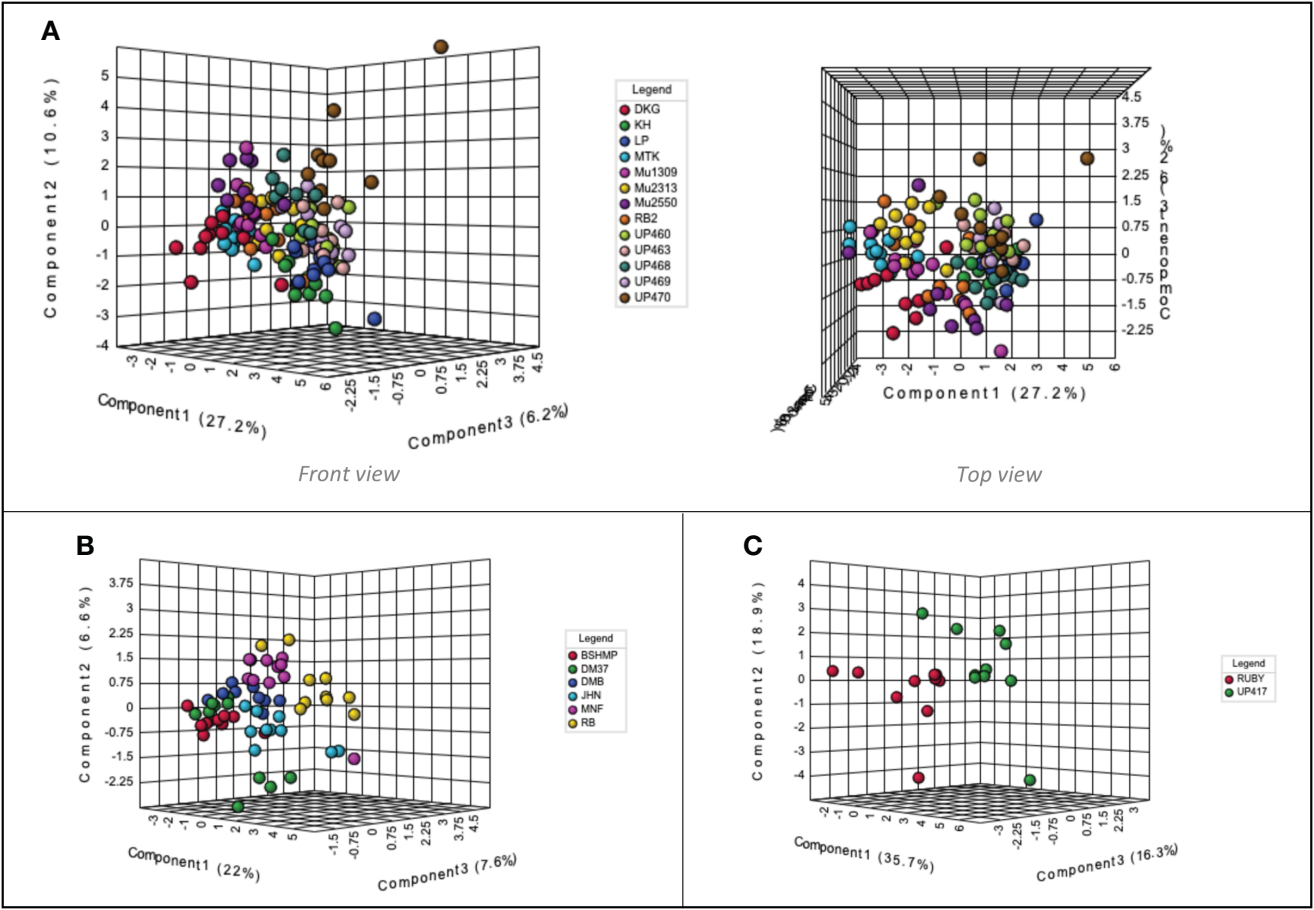
Partial least squares-discriminant analysis (PLS-DA) scores plot of Thai colored rice volatile profiles identified by static headspace gas chromatography–mass spectrometry technique. **(A)** aromatic black rice cultivars, **(B)** non-aromatic black rice cultivars, **(C)** non-aromatic red rice cultivars. One replication is represented by each colored symbol (n=10 per rice cultivar), while 95% confidence intervals are represented by shaded circles.

When considering each colored rice sample group independently, the volatile compounds present in black aromatic rice varieties demonstrates minimal separation. Nevertheless, from the top view, the volatile components of UP460, UP463, UP468, UP469, and UP470 are close to each other and positioned slightly isolated from DKG, MTK, Mu1309, Mu2313, Mu2550, and RB2 (**Figure 4A**). As for the volatile components of non-aromatic black rice cultivars, the RB aroma profile is related to MNF. However, it is quite different from that of BSHMP, located close to DM37 and DMB (**Figure 4B**). The red rice volatile profiles RUBY and UP417 are plotted separately in the PLS-DA scores plot (**Figure 4C**).

**Figure 5:**
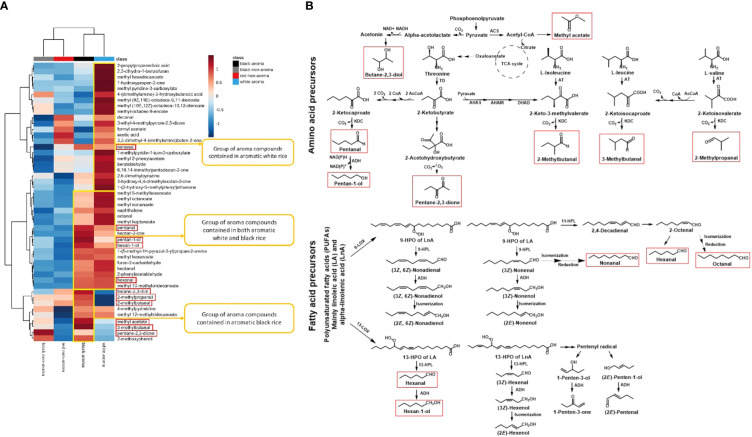
**(A)** Hierarchical clustering heatmaps of volatile compounds identified in aromatic white, aromatic black, non-aromatic black, and non-aromatic red rice samples. Warm color (red) indicates an increase of volatile compounds while cold color (blue) indicates a decrease of volatile chemical levels. The key and main volatile products detected in Thai black-aroma rice are highlighted by red-square frames which was proposed to be biosynthesized *via* the pathways summarized in **(B)**. **(B)** The proposed biosynthetic pathways in Thai aroma-black rice of various volatile aldehydes and alcohols starting from related fatty acid and branched-chain amino acid precursors. The key and main volatile products detected in Thai black-aroma rice highlighted in A are also highlighted with the same red-square frames. LOX; lipoxygenase, HPL; hydroperoxide lyase, ACS; Acetyl-CoA synthetase, TD; threonine dehydratase, AT; aminotransferase, AHAS; acetohydroxyacid synthase, AHAIR; acetohydroxyacid isomeroreductase, DHAD; dihydroxyacid dehydratase, KDC; 2-keto acid decarboxylases and ADH; alcohol dehydrogenases.

Heatmap hierarchical cluster analysis (HCA) of volatile metabolomics data provide a simplify data visualization (**Figure 5A**). Overall, the hierarchical clustering heatmap with used of Euclidean distances and the Ward method reveals the different patterns of volatile chemicals derived from the various colored rice groups. The yellow boxes denote groups of aroma compounds mainly found in each type of rice. There are 22, 17 and 9 compounds primarily present in white aroma, both white & black aroma, and black aroma rice groups, respectively. The key and main volatile products detected in Thai black-aroma rice are highlighted by red-square frames which were proposed to be biosynthesized *via* the pathways summarized in **Figure 5B**.

**Figure 6:**
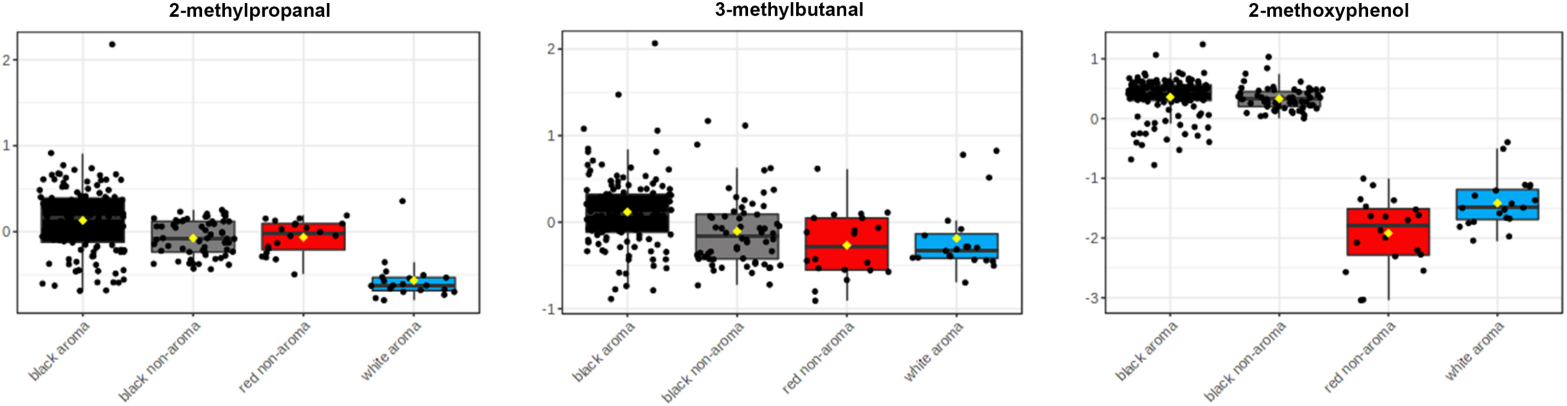
Box and whisker plots of principal volatile compounds identified in Thai aromatic black rice cultivars (black bar) as compared to aromatic white rice (blue bar), non-aromatic black rice (gray bar), and non-aromatic red rice groups (red bar).

According to heatmap and statistical analysis in **Table 2**, the three main volatile compounds found in the black rice samples as compared to the four categories of rice are 2-methylpropanal, 3-methylbutanal, and 2-methoxyphenol as shown in **Figure 6**. Levels of 2-methylpropanal were greater in the colored rice than the white rice. Black aroma rice had the highest concentrations of 3-methylbutanal compared to other rice groups. Black rice cultivars (both aroma and non-aroma) had higher amounts of 2-methoxyphenol than white and red rice.

**Figure 7:**
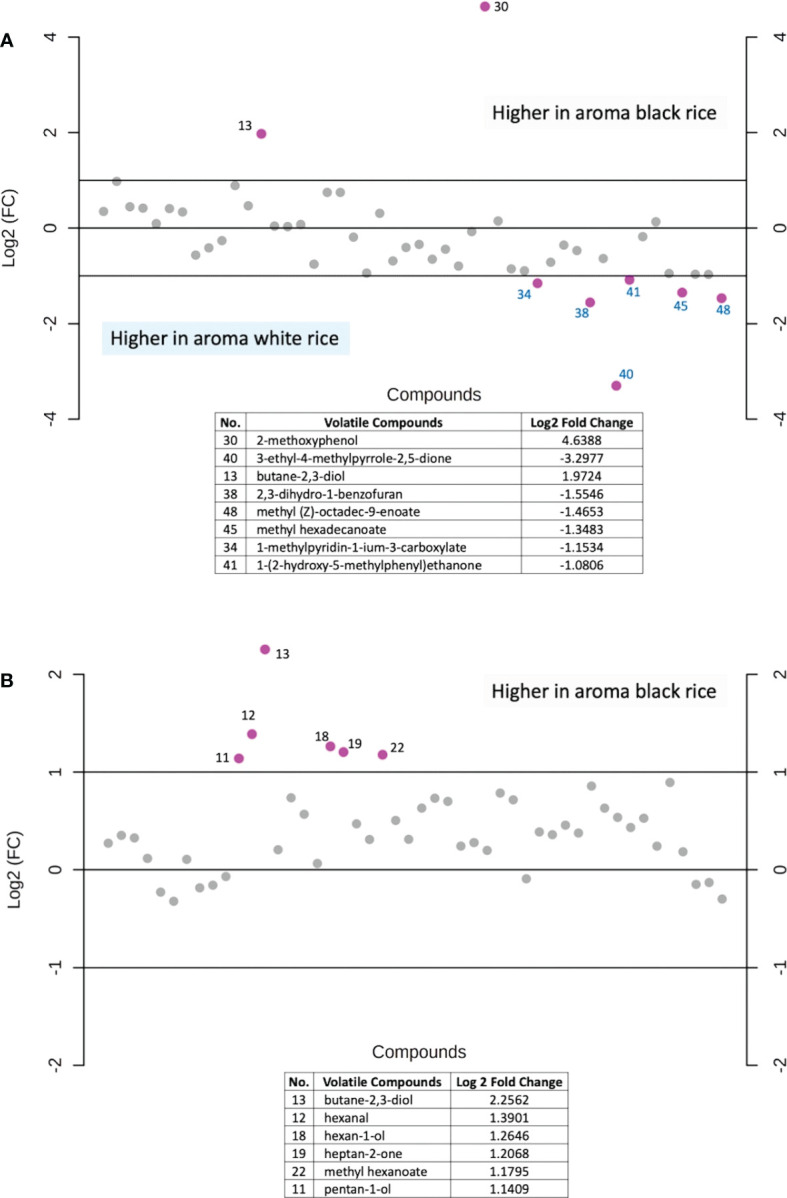
Fold-change analysis of the volatile compounds abundance between **(A)** aromatic black rice and aromatic white rice samples, **(B)** aromatic black rice and non-aromatic black rice samples. Purple dot represents each rice volatile compounds with a two-fold changes (increase or decrease). Each number represented the identified volatile compounds arranged by retention time.

In addition, fold-change values were calculated to identify which volatile compounds are more abundant between the two rice groups (**Figure 7**). When compare between aroma rice (aroma black vs. aroma white rice), the results illustrate that white rice has a higher concentration of several volatile components while there are only two substances, 2-methoxyphenol and butane-2,3-diol, that are higher in the black fragrant rice (**Figure 7A**). On the contrary, when comparing solely the two types of black rice (aroma vs. non-aroma), aroma black rice exhibits higher levels of many volatile compounds than the non-aroma rice (**Figure 7B**).

## 4 Discussion

### 4.1 Volatile components in Thai colored rice cultivars

The colored rice samples in this study were obtained from landrace rice varieties from the Rice Research Center, Thailand’s Rice Department as well as new rice varieties from crossbreeding and induced mutagenesis developed by the Rice Science Center, Kasetsart University, Thailand, creating unique rice aroma that may differ from various cultivate locations. The results showed that the main volatile found in the black rice was 2-methoxyphenol. Although both 2-methoxyphenol and butane-2,3-diol showed large fold-changes when compared between the two aroma rice groups (black vs. white, **Figure 7A**), levels of butane-2,3-diol were not significant different among the four rice groups as revealed in **Table 2**. 2-methoxyphenol levels in both aromatic and non-aromatic black rice were significantly higher than in white and red rice (p<0.0001), with the highest f-value (393.94) and the highest VIP score (>2.0). This finding agrees with previous research by Yang et al. (2008), who found that 2-methoxyphenol is the primary component underlying black rice’s uniqueness. 3-methylbutanal was highly present in aromatic black rice as compared to other rice types. This compound was also reported as volatile in cooked black rice (Song et al., 2000). 2-methylpropanal, a volatile chemical present in numerous foods, was another volatile substance found at lower concentration in white rice than in black and red rice (**Figure 6**).

In aromatic rice samples with BADH2 genotype, volatile compounds detected in both white and black aromatic rice unique to non-aromatic rice in this study were methyl 5-methylhexanoate, methyl octanoate, 4-methylpyrimidine, methyl hexanoate, methyl nonanoate, hexanal, methyl 10-methylundecanoate, heptan-2-one, octanal, hexan-1-ol, naphthalene, furan-2-carbaldehyde, 1-(5-methyl-1H-pyrazol-3-yl)propan-2-amine, pentan-1-ol, nonanal, and 2-phenylacetaldehyde (**Figure 5A**). Nevertheless, several volatile components in white fragrant rice samples were found at higher levels than in aromatic black rice samples. These aroma compounds include 3-ethyl-4-methylpyrrole-2,5-dione, previously observed in pandan leaves (Cheetangdee and Chaiseri, 2006); 2,6-dimethylpyrazine, that gives a bread-like aroma (FooDB, 2022); 1-(2-hydroxy-5-methylphenyl)ethanone and 3,3-dimethyl-4-(methylamino)butan-2-one, with a sweet floral fragrance (Koksal et al., 2015; FooDB, 2022); benzaldehyde and methyl 2-phenylacetate, a methyl ester with an almond-like smell (FooDB, 2022); 2-propylpropanedioic acid, found in honey (Tian et al., 2018); 1-methylpyridin-1-ium-3-carboxylate or trigonelline, found in roasted coffee (Heo et al., 2020; FooDB, 2022); and the characteristic tobacco-like herbaceous odor of methyl pyridine-3-carboxylate or methyl nicotinate (Rao et al., 2007; FooDB, 2022).

In addition, many rice-related volatile compounds were found at higher amounts in white rice samples. These compounds were 2,3-dihydro-1-benzofuran, contained in the rice husks (Tian et al., 2021); 3-hydroxy-4,4-dimethyloxolan-2-one, formerly observed in cooked rice (Jinakot and Jirapakkul, 2018); and 6,10,14-trimethylpentadecan-2-one, the major volatile substance of red rice (Sukhonthara et al., 2009), found in high concentrations in both white and red rice samples in this study. Fatty aldehydes such as decanal as well as fatty acid methyl esters including methyl-octadec-9-enoate, methyl hexadecanoate, and methyl (10E,12Z)-octadeca-10,12-dienoate were also identified.

Unsurprisingly, when only the black variety is considered, aroma black rice contains more volatile compounds than the non-aroma rice (**Figure 7B**). Volatile substances that have been reported pleasant smells include a buttery, creamy scent from butane-2,3-diol (FooDB, 2022); a fruity and floral-like smell from heptan-2-one (Verma and Srivastav, 2020); and a sweet, fresh flavor from methyl hexanoate (FooDB, 2022). Hexanal and hexan-1-ol contribute to a green scent in rice (Verma and Srivastav, 2020; Choi and Lee, 2021); and pentan-1-ol is described a fusel oil-like odor (Verma and Srivastav, 2020), which might contribute to the unpleasant smell of the black rice.

### 4.2 Key volatile compounds and related biosynthetic pathways

From the hierarchical clustering heatmaps shown in **Figure 5A** which summarizes quantitatively various volatile components detected in the four categories of rice samples, it can be seen that, in general, the non-aroma group (black and red) contained much lower content of most volatile components than that of the aroma group (black and white). Interestingly, the heatmap also clearly shows that each rice category has its own uniqueness in terms of major volatile components. The non-aroma black rice showed high content of pentane-2,3-dione, 2-methoxyphenol and 4-methylpyrimidine while the non-aroma red showed high content of acetic acid, decanal, 3,3-dimethyl-4-(methylamino)butan-2-one. On the other hand, the aroma black appeared to contain high content of some aldehyde components, specifically of 3-methylbutanal, 2-methylbutanal, 2-methylpropanal, pentanal, hexanal, and some alcohol components, mainly of butane-2,3-diol, pentan-1-ol, and hexan-1-ol (**Figure 5A**, column 3 from the left highlighted with red squares). By comparing with the white-aroma rice, it can be seen that these main black-aroma constituents are in only the minor components of the white-aroma rice. This suggested that the biosynthetic pathways responsible for the formation of these volatile components were operated at different flow rates among the four different rice categories.

In order to understand the observed characteristic patterns of the volatile components in various rice samples, biosynthetic pathways possibly involved in the formation of these compounds were introduced to explain the results. The black-aroma rice which contains both unique and common volatile constituents was used as a working model for this purpose. As shown in **Figure 5B**, we proposed that the biosynthetic pathways that utilize amino acids and polyunsaturated fatty acids are the two main routes that produce the fragrant compounds in the black-aroma rice. In this case, the amino acid precursors were proposed to be L-threonine, L-isoleucine, L-leucine, and L-valine, and the polyunsaturated fatty acid precursors were linoleic acid and α-linolenic acid. The four amino acid precursors are likely to be metabolized through their degradative pathways to form their corresponding 4-6 carbon components of the aldehyde and alcohol compounds while the two polyunsaturated fatty acids can be metabolized through the action of 9-lipoxygonase and 13-lipoxygease enzymes to form the longer 6-10 carbon chains of the observed aldehyde and alcohol products (**Figure 5B**). Specifically, L-threonine is metabolized to form pentanal and pentan-1-ol, L-isoleucine to 2-methylbutanal, L-leucine to 3-methylbutanal, L-valine to 2-methylpropanal, linoleic acid to hexanal and hexan-1-ol, and α-linolenic acid to hexanal, nonanal and octanal. By analyzing the quantitative heatmap data (**Figure 5A**) based on the proposed biosynthetic pathways (**Figure 5B**), it is possible to explain the difference between the aroma and non-aroma rice in terms of their biosynthetic gene expression. For example, the content of the mentioned amino acid-derived aldehyde volatiles which showed higher in the black-aroma rice than in the non-aroma and the white rice might be due to the higher gene expression in the black-aroma of various enzymes of aminotransferases (ATs) and 2-keto acid decarboxylases (KDCs). Both groups of ATs and KDCs are responsible for the conversion of the four amino acid precursors to the detected aldehyde products. Similarly, the observed high levels of the fatty acid-derived aldehyde (hexanal) and alcohol (hexane-1-ol) in the same black-aroma rice might also be due to high gene expression of their related hydroperoxide lyase (HPL) and alcohol dehydrogenase (ADH), respectively (**Figure 5B**). It has been reported that the polyunsaturated fatty acids can undergo oxidation *via* the lipoxygenase pathway to produce aliphatic alcohols, aldehydes, methyl ketones, and esters (Siegmund, 2015). Saturated fatty acids, on the other hand, use the β-oxidation pathway to generate oxygenated aliphatic hydrocarbons (Wong et al., 2017). Amino acid degradation could produce methyl-branched alcohols, aldehydes, acids, and esters. First, the decarboxylase enzyme converts amino acids to amines. Aminotransferase can also convert amino acids to 2-keto acids, resulting in aldehydes, alcohols, and acids as end products (Schwab et al., 2008; Langyan et al., 2022).

In terms of scented aroma, the distinctive volatile compounds of 3-methylbutanal, 2-methylbutanal, methyl acetate, and butane-2,3-diol are likely to contribute to its unique smell in black-aroma rice. Particularly, 3-methylbutanal and 2-methylbutanal, the components of cocoa’s aroma (Frauendorfer and Schieberle, 2006), could be the dominant smell of the aromatic black rice group. Both aldehydes, again, originate from two closely related amino acids of leucine and isoleucine (Kochevenko et al., 2012). These two amino acids are primary converted to α-keto-acids by branched-chain aminotransferases, then the aldehydes are produced by the 2-keto-acid decarboxylase, and finally alcohols are produced by the aldehyde dehydrogenase. As a result, the 2-keto-acid decarboxylase gene is most likely the major gene associated with the volatile compounds in black rice, as it is directly responsible for the production of these volatile molecules.

## 5 Conclusion

The key volatile aromas in Thai native-colored rice cultivars were identified using SHS-GC-MS untargeted metabolomics approach. 2-methylpropanal was the most distinctive volatile in colored rice (black and red rice). 2-methoxy phenol was mainly found in both aromatic and non-aromatic black rice, while 3-methylbutanal was the major compound in aromatic black rice. The precursors of these main unique volatile chemicals in fragrant black rice samples could be leucine and isoleucine. Branched-chain aminotransferases, followed by keto-acid decarboxylases are the key enzymes responsible for precursor conversion to the volatile products. The final products of these proposed pathways appeared to agree well with the volatile components found in the black-aroma rice samples, supporting the correct biosynthetic pathways proposed to be involved in the volatile compound formation in the black-aroma rice. However, it should be noted that all the volatile constituents were detected in all the four rice categories but in different accumulated contents, we proposed that the unique scented aroma of each type of rice would depend on the different metabolic rates operated by the same pathways rather than by different ones. The factors controlling this complex metabolism remain to be determined.

## Data availability statement

The original contributions presented in the study are included in the article/**Supplementary Materials**. Further inquiries can be directed to the corresponding authors.

## Author contributions

Conceptualization and grant finding were done by WD-E, AV and SV. RT and WD-E designed the experiment and drafted the main manuscript. Rice planting and seeds were prepared by SR and AV. GC-MS metabolomic analysis was carried out by SJ, PE, and NS. Data management was done by RT and PE. RT performed statistical analysis. All authors contributed to the article and approved the submitted version.
