# Supplementary figures and images for: Metabolomics approach to identify key volatile aromas in Thai colored rice cultivars
Source: Front Plant Sci. 2023 Feb 28;14:973217. doi: 10.3389/fpls.2023.973217 (PMC10011493; doi:10.3389/fpls.2023.973217)

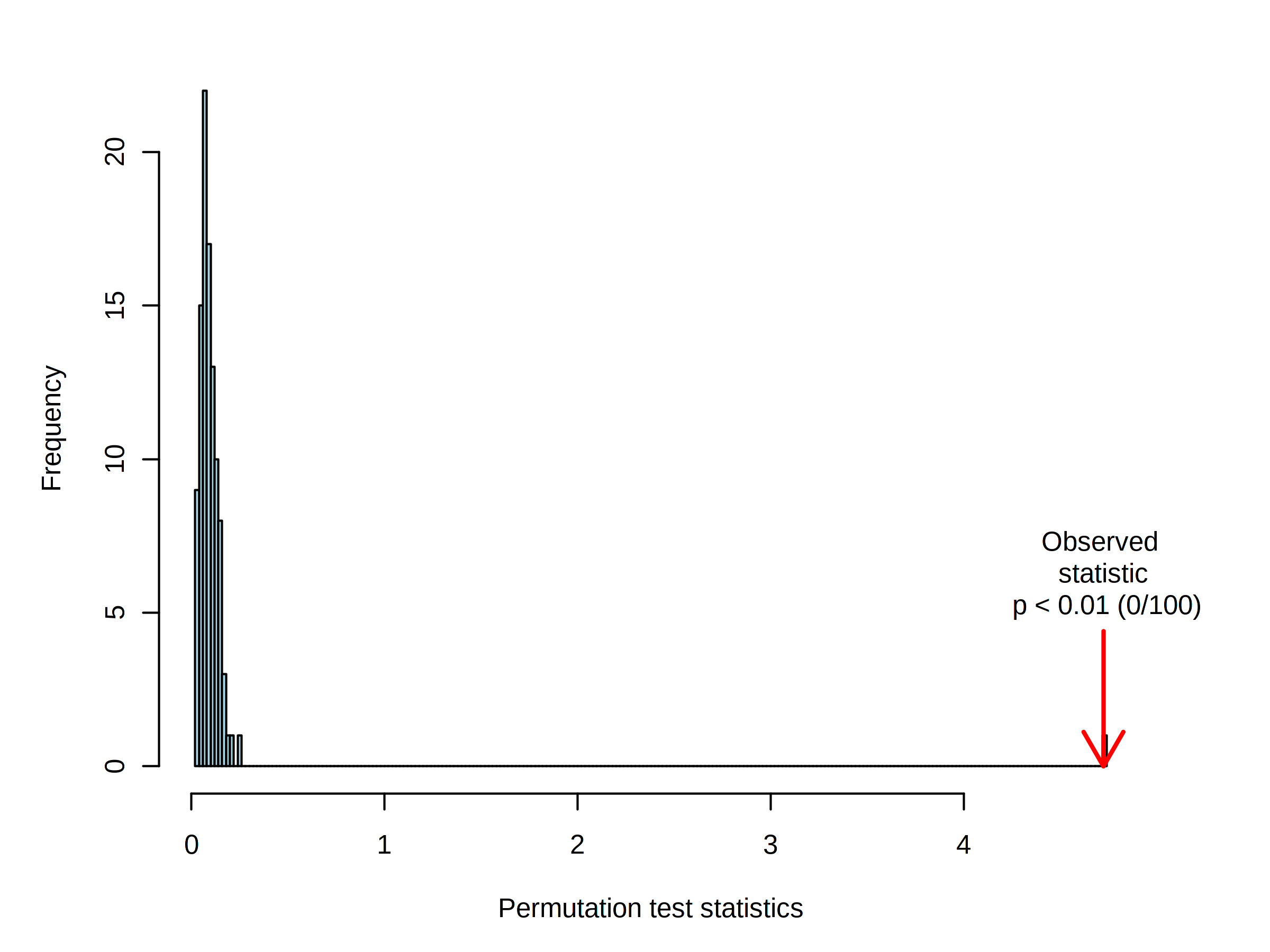

Supplement: Supplementary file 2 [file Image_1.tiff]
